# Early-phase cumulative hypotension duration and severe-stage progression in oliguric acute kidney injury with and without sepsis: an observational study

**DOI:** 10.1186/s13054-016-1564-2

**Published:** 2016-12-19

**Authors:** Junichi Izawa, Tetsuhisa Kitamura, Taku Iwami, Shigehiko Uchino, Masanori Takinami, John A. Kellum, Takashi Kawamura

**Affiliations:** 1 Department of Preventive Services, Kyoto University School of Public Health, Yoshida-Honmachi, Sakyo-ku, Kyoto, 606-8501 Japan; 2 Intensive Care Unit, Department of Anesthesiology, The Jikei University School of Medicine, 3-19-18 Nishi-Shinbashi, Minato-ku, Tokyo, 105-8471 Japan; 3 Division of Environmental Medicine and Population Sciences, Department of Social and Environmental Medicine, Graduate School of Medicine, Osaka University, 1-1 Yamada-oka, Suita, Osaka, 565-0871 Japan; 4 Center for Critical Care Nephrology, University of Pittsburgh Medical Center, 604 Scaife Hall, 3550 Terrace Street, Pittsburgh, PA 15261 USA

**Keywords:** Critical illness, Acute kidney injury, Arterial pressure, Hypotension, Intensive care unit, Epidemiology

## Abstract

**Background:**

Managing blood pressure in patients with acute kidney injury (AKI) could effectively prevent severe-stage progression. However, the effect of hypotension duration in the early phase of AKI remains poorly understood. This study investigated the association between early-phase cumulative duration of hypotension below threshold mean arterial pressure (MAP) and severe-stage progression of oliguric AKI in critically ill patients, and assessed the difference in association with presence of sepsis.

**Methods:**

This was a single-center, observational study conducted in the ICU of a university hospital in Japan. We examined data from adults with oliguric AKI who were admitted to the ICU during 2010–2014 and stayed in the ICU for ≥24 h after diagnosis of stage-1 oliguric AKI defined in the Kidney Disease Improving Global Outcomes (KDIGO) guidelines. The primary outcome was the progression from stage-1 oliguric AKI to stage-3 oliguric AKI (progression to oligoanuria and use of renal replacement therapy) according to the KDIGO criteria. During the first 6 h after oliguric AKI, we analyzed the association between cumulative time the patient had below threshold MAP (65, 70, and 75 mm Hg) and progression to stage-3.

**Results:**

Among 538 patients with oliguric AKI, progression to stage-3 increased as the time spent below any threshold MAP was elongated. In the multivariable analysis of all patients, longer hypotension time (3–6 h) showed significant association with stage-3 progression for the time spent below MAP of 65 mm Hg (adjusted odds ratio (OR) 3.73, 95% confidence interval (CI) 1.53–9.09, *p* = 0.004), but the association was attenuated for the threshold MAP of 70 mm Hg (adjusted OR 2.35, 95% CI 0.96–5.78, *p* = 0.063) and 75 mm Hg (adjusted OR 1.92, 95% CI 0.72–5.15, *p* = 0.200). Longer hypotension time with the thresholds of 65 and 70 mm Hg was significantly associated with the risk of stage-3 progression in patients without sepsis, whereas the association was weak and not significant in patients with sepsis.

**Conclusions:**

Even in a short time frame (6 h) after oliguric AKI diagnosis, early-phase cumulative hypotension duration was associated with progression to stage-3 oliguric AKI, especially in patients without sepsis.

## Background

Acute kidney injury (AKI) is an extremely common burden in intensive care units (ICUs) [1]. When critical illness is complicated by AKI, there is a well-known association with increased mortality [1, 2], but preventive measures have not yet been established. Maintenance of normal or even increased mean arterial pressure (MAP) could provide a benefit because autoregulation of blood flow is known to be lost during certain forms of AKI, especially in patients with sepsis [3]. Recent studies [4, 5] suggest that maintaining MAP ≥70 mm Hg could better protect patients with sepsis and AKI compared to maintaining MAP ≥65 mm Hg as recommended in the worldwide guidelines for sepsis [6, 7]. Other studies indicate that the time elapsed with blood pressure below threshold might be the key indicator of developing postoperative AKI [8, 9]. On the other hand, the optimal blood pressure threshold is still a matter of debate. Some studies have failed to indicate the effectiveness of maintaining high MAP in AKI management [10, 11].

Blood pressure management strategies to prevent progression of AKI to severe stages need to be established. Some reports exhibit the association between the time spent with blood pressure below threshold MAP and progression of AKI in patients with sepsis [12, 13]. However, whether intensivists should also manage blood pressure in patients with AKI without sepsis remains a point of contention [4, 5, 12–14]. Moreover, it is uncertain whether managing hypotension duration in early-phase AKI is important in preventing progression to severe-stage AKI.

This study focused on oliguria, an easy-to-find marker for early-stage AKI in the ICU. In the early phase of oliguric AKI (6 h after the diagnosis of oliguric AKI), we investigated the association between cumulative hypotension duration below threshold MAP and progression of AKI among critically ill patients with and without sepsis.

## Methods

### Study design, setting, and patients

This observational study reviewed data from all consecutive patients admitted to the ICU of the Jikei University School of Medicine, Tokyo, Japan, from January 2010 through December 2014. The study protocol was approved by the respective medical institutional review boards of Kyoto University (approval number R0432) and Jikei University (approval number 27-299). Because of the retrospective approach of this study and de-identification of personal data, the boards waived the need for informed consent.

We examined data from consecutive patients aged ≥18 years who had not undergone maintenance dialysis and who stayed in the ICU for at least 24 h after the diagnosis of oliguric AKI, which was defined as stage-1 oliguric AKI, urine output <0.5 mL/kg/h for 6 h consecutively (Table 1), according to the Kidney Disease Improving Global Outcomes (KDIGO) criteria [15]. If patients with oliguric AKI were admitted to the ICU twice or more during the study period, only data from the first ICU admission were included. We excluded patients in whom blood pressure was not measured with an interval of <1 h during the 24-h period after oliguric AKI diagnosis, and those who died within 72 h after ICU admission. Patients who had been admitted after vascular surgery were excluded because they were frequently treated with higher target levels of blood pressure in the ICU to avoid spinal ischemia and paraplegia [16]. We also excluded patients in whom renal replacement therapy (RRT) was initiated within 6 h of oliguric AKI diagnosis.

**Table 1:** Staging of acute kidney injury for adults according to KDIGO

Staging	Serum creatinine	Urine output
Stage 1	1.5–1.9 times baseline, or ​≥0.3 mg/dL (≥26.5 μmol/L) increase	<0.5 mL/kg/h for 6–12 h^a^
Stage 2	2.0–2.9 times baseline	<0.5 mL/kg/h for ≥12 h
Stage 3	3.0 times baseline, or increase in serum creatinine to ≥4.0 mg/dL (≥353.6 μmol/L), or initiation of renal replacement therapy	<0.3 mL/kg/h for ≥24 h^b^, or anuria for ≥12 h^b^

Acute kidney injury (AKI) is defined as any of the following: (1) increase in serum creatinine by ≥0.3 mg/dL (≥26.5 μmol/L) within 48 h; (2) increase in serum creatinine by ≥1.5 times baseline, which is known or presumed to have occurred within the prior 7 days; (3) urine output <0.5 mL/kg/h for 6 h. AKI was diagnosed according to the Kidney Disease Improving Global Outcomes (KDIGO). ^a^Definition of stage-1 oliguric AKI in this study. ^b^Definition of stage-3 oliguric AKI (progression to oligoanuria and use of renal replacement therapy) in this study

### Data collection

Data for analyses including age, sex, body weight, comorbidities, ward type before ICU admission, surgery type for postoperative patients, prevalence of sepsis at ICU admission, length of ICU stay, ICU mortality, and hospital mortality were collected from the ICU database. In this study, sepsis was defined as the presence of known active systemic infection at ICU admission, the presence of shock at ICU admission caused by suspected infection, or positive blood culture sampled at ICU admission. We also collected data on urine output for 7 days after ICU admission or until ICU discharge, blood pressure, and intravenous administration of vasoactive agents (dopamine, dobutamine, norepinephrine, and vasopressin) for 6 h after oliguric AKI diagnosis via the electronic medical record system (PIMS; Philips Japan Ltd.). The time between ICU admission and oliguric AKI diagnosis (time to oliguric AKI) was calculated.

Illness severity was assessed using Acute Physiology and Chronic Health Evaluation (APACHE) II scores [17] and Sequential Organ Failure Assessment (SOFA) scores [18] during the first 24-h period after ICU admission. In the ICU, urine output was recorded every 2 h. The MAP was recorded every 15 minutes. We collected fluid balance data, but the time to obtain the fluid balance information was predefined at three points per day (at 5:00, 13:00 and 21:00 h) in the ICU. Therefore, we obtained fluid balance data during the first measurable 8-h period after oliguria started. Although we also obtained serum creatinine at ICU admission, we did not collect baseline serum creatinine data because our prior research shows that more than half of the patients in the ICU did not have baseline creatinine recorded [19].

### Outcome measures

The primary outcome measure was progression to stage-3 oliguric AKI (progression to oligoanuria and use of renal replacement therapy (RRT)) according to the KDIGO criteria: RRT initiation during the stay in the ICU, urine volume <0.3 mL/kg/h for 24 h consecutively within 7 days after ICU admission, or anuria for 12 h consecutively within seven days after ICU admission (Table 1) [15]. We did not use the criteria for serum creatinine in the KDIGO guidelines, because we specifically examined oliguria as the marker for early diagnosis of AKI. We additionally performed sensitivity analyses by focusing on initiation of RRT as the outcome.

### Measure of main exposure factors

To assess the association between blood pressure and the progression to stage-3 oliguric AKI, we considered the cumulative hypotension duration as the most clinically relevant variable of blood pressure parameters based on recent studies [8, 9]. Here, we set three hypotension thresholds: MAP of 65, 70, and 75 mm Hg. The cumulative time below a particular threshold MAP in a 6-h period after oliguric AKI diagnosis was considered as the main exposure factor for stage-3 progression. Stage-3 progression according to the urine output criteria is possible at 6 h after the oliguric AKI diagnosis. Therefore, we stopped data collection on exposure at 6 h after oliguric AKI diagnosis. We also calculated the “time-averaged MAP,” “lowest MAP” and “area under threshold MAP” in the 6-h period.

### Statistical analysis

Data were analyzed as medians with interquartile range (IQR) for continuous variables and as proportions for categorical variables, to which the Mann–Whitney *U* test and Fisher's exact test or chi-squared test were applied, respectively. We first visually described the relationship between the cumulative time spent below each threshold MAP (65, 70, and 75 mm Hg) and progression to stage-3 AKI using restricted cubic splines in univariable logistic regression models. To evaluate the predictability of each blood pressure parameter for stage-3 progression, we drew the area under the receiver operating curve (AUROC).

As the primary analysis, multivariable logistic regression models were used to assess the association between the time categories below each threshold MAP and stage-3 progression. Here, the time category was divided into none (0 hours), minimal through 3 h (0–3 h) and 3 through 6 h (3–6 h), and the odds ratios (ORs) and the 95% confidence intervals (CIs) were calculated. The following variables were incorporated into the primary multivariable models: serum creatinine levels at ICU admission, the prevalence of sepsis at ICU admission, and APACHE II scores. Furthermore, we performed subgroup analyses stratified by sepsis and non-sepsis to elucidate the differences in etiology. All statistical analyses were performed using R (The R Foundation for Statistical Computing, ver. 3.30) and EZR (Saitama Medical Center, Jichi Medical University, ver. 1.32), which is a graphical user interface for R [20]. All tests were two-tailed; *p* values <0.05 were regarded as statistically significant.

## Results

**Fig. 1:**
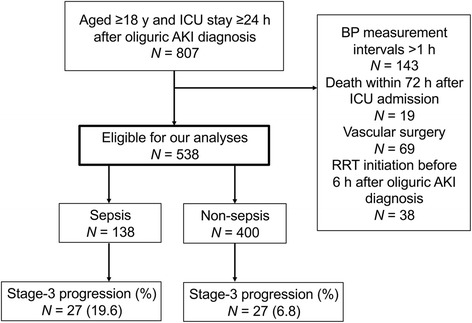
Flow of patients with oliguric acute kidney injury (*AKI*) admitted to the ICU from January 2010 through December 2014. *BP* blood pressure, *RRT* renal replacement therapy

The selection process for the study patients is presented in Fig. 1. We extracted 807 patients who stayed in the ICU for ≥24 h after oliguric AKI diagnosis. After excluding 269 patients who met the aforementioned exclusion criteria, we enrolled 538 patients with stage-1 oliguric AKI for our analyses. Progression to stage 3 was observed in 27 (19.6%) of 138 patients with sepsis and 27 (6.8%) of 400 patients without sepsis at ICU admission.

Patient characteristics, vasoactive use and fluid balance are presented in Table 2. Among the patients, 51.7% (278/538) were admitted to the ICU after surgery; 25.7% (138/538) had sepsis at ICU admission. The median APACHE II score was 17 (IQR 13–22). Patients with stage-3 progression had significantly higher serum creatinine levels at ICU admission (1.54 mg/dL vs. 0.86 mg/dL, *p* < 0.001), shorter time to oliguric AKI (10.7 h vs. 19.0 h, *p* = 0.001), higher SOFA scores (11 vs. 7, *p* < 0.001), and higher APACHE II scores (22 vs. 17, *p* < 0.001). During the 6-h period after oliguric AKI diagnosis, patients with stage-3 progression had more frequently received norepinephrine (66.7% (36/54) vs. 42.4% (205/48), *p* = 0.001) and vasopressin (11.1% (6/54) vs. 2.9% (14/484), *p* = 0.010) than patients without progression. During the first measurable 8-h period after the start of oliguria, patients with stage-3 progression had more fluid input (949 mL vs. 758 mL, *p* = 0.004), less fluid output (272 mL vs. 326 mL, *p* = 0.028) and more positive fluid balance (723 mL vs. 432 mL, *p* < 0.001) than in patients without progression. In patients with sepsis, however, the fluid parameters were similar between groups of patients with stage-3 progression and those with no progression (input: 848 mL vs. 840 mL, *p* = 0.265; output: 281 mL vs. 262 mL, *p* = 0.979; balance: 514 mL vs. 470 mL, *p* = 0.551). Of all patients, 6.7% (36/538) and 15.6% (84/538) died in the ICU and in hospital, respectively.

**Table 2:** Patient characteristics, vasoactive use and fluid balance in ICU patients with oliguric AKI

	Stage-3 progression	No progression	*P* value
	(n = 54)	(n = 484)	
*Characteristics*			
Age (years; median (IQR))	69 (61–78)	70 (59–77)	0.647
Male (%)	41 (75.9)	335 (69.2)	0.351
Body weight (kg; median (IQR))	62.0 (52.3–67.8)	60.0 (50.0–70.0)	0.571
Comorbidities (%)			
Immunocompromised	8 (14.8)	42 (8.7)	0.141
Hematologic malignancy	2 (3.7)	12 (2.5)	0.642
Metastasis	0 (0.0)	14 (2.9)	0.380
Respiratory failure	3 (5.6)	19 (3.9)	0.476
Heart failure	0 (0.0)	2 (0.4)	1.000
Liver failure	3 (5.6)	8 (1.7)	0.088
Ward type before ICU admission (%)			
Operating room	18 (33.3)	260 (53.7)	0.008
Cardiac surgery	10 (18.5)	127 (26.2)	
Thoracic surgery	2 (3.7)	42 (8.7)	
Neurosurgery	0 (0.0)	31 (6.4)	
Other major surgery	6 (11.1)	60 (12.4)	
General ward	21 (38.9)	109 (22.5)	
Emergency department	15 (27.8)	115 (23.8)	
Sepsis at ICU admission (%)	27 (50.0)	111 (22.9)	<0.001
Serum creatinine at ICU admission (mg/dL; median (IQR))	1.54 (1.04–3.10)	0.86 (0.67–1.19)	<0.001
Time to oliguric AKI (hours; median (IQR))	10.7 (7.4–28.5)	19.0 (10.7–35.5)	0.001
APACHE II score (median (IQR))	22 (17–27)	17 (13–21)	<0.001
SOFA score (median (IQR))	11 (8–13)	7 (4–9)	<0.001
Respiratory	2 (1–3)	2 (1, 2)	0.182
Coagulation	2 (0–2)	1 (0–2)	0.009
Liver	1 (0–1)	0 (0–1)	0.098
Cardiovascular	4 (1–4)	3 (1–4)	0.040
Central nervous system	0 (0–1)	0 (0–1)	0.130
Renal	3 (1–3)	0 (0–1)	<0.001
*Vasoactive use within 6 h after oliguric AKI diagnosis*			
Dopamine (%)	0 (0.0)	10 (2.1)	0.609
Dobutamine (%)	9 (16.7)	97 (20.0)	0.718
Norepinephrine (%)	36 (66.7)	205 (42.4)	0.001
Vasopressin (%)	6 (11.1)	14 (2.9)	0.010
*Fluid balance during the first measurable 8-h period after oliguria starting*			
In (mL; median (IQR))	949 (691–1526)	758 (530–1096)	0.004
Out (mL; median (IQR))	272 (158–413)	326 (200–506)	0.028
Balance (mL; median (IQR))	723 (386–1278)	432 (179–759)	<0.001

Time to oliguric acute kidney injury (*AKI*), time between ICU admission and oliguric AKI diagnosis. Sequential Organ Failure Assessment (*SOFA*) scores were calculated using information during the first 24-h period after ICU admission. *APACHE* Acute Physiology and Chronic Health Evaluation, *IQR* interquartile range

Blood pressure parameters in patients with stage-3 progression and those with no progression are presented in Table 3. Among all patients, patients with stage-3 progression exhibited significantly lower time-averaged MAP (71 mm Hg vs. 75 mm Hg, *p* = 0.003), lower lowest MAP (61 mm Hg vs. 65 mm Hg, *p* < 0.001), larger area under any threshold MAP (65 mm Hg: 2.0 h × mm Hg vs. 0.0 h × mm Hg, *p* = 0.001; 70 mm Hg: 10.0 h × mm Hg vs. 3.3 h × mm Hg, *p* = 0.001; 75 mm Hg: 27.8 h × mm Hg vs. 14.4 h × mm Hg, *p* = 0.001), and longer time below any threshold MAP (65 mm Hg: 0.5 h vs. 0.0 h, *p* = 0.002; 70 mm Hg: 2.4 h vs. 1.0 h, *p* = 0.002; 75 mm Hg: 4.6 h vs. 2.8 h, *p* = 0.002) than patients without progression. The AUROC of time-averaged MAP and lowest MAP was 0.62 (95% CI 0.54–0.71) and 0.63 (95% CI 0.55–0.71), respectively, for all patients. The AUROC of area under threshold MAP was 0.63 (95% CI 0.55–0.71) in 65 mm Hg, 0.63 (95% CI 0.55–0.71) in 70 mm Hg and 0.64 (95% CI 0.56–0.71) in 75 mm Hg. For the AUROC of time below threshold MAP, the AUROC was 0.62 (95% CI 0.54–0.70) in 65 mm Hg, 0.63 (95% CI: 0.55–0.70) in 70 mm Hg and 0.63 (95% CI: 0.55–0.71) in 75 mm Hg.

**Table 3:** Blood pressure parameters within 6 h after oliguric AKI diagnosis

	All patients (n = 538)			Sepsis (n = 138)			Non-sepsis (n = 400)		
	Stage-3 progression	No progression	*P* value	Stage-3 progression	No progression	*P* value	Stage-3 progression	No progression	*P* value
	(n = 54)	(n = 484)		(n = 27)	(n = 111)		(n = 27)	(n = 373)	
Time-averaged MAP (mm Hg (IQR))	71 (65–77)	75 (70–83)	0.003	73 (70–81)	75 (71–83)	0.356	70 (62–74)	75 (69–83)	0.001
Lowest MAP (mm Hg (IQR))	61 (55–67)	65 (60–73)	0.002	62 (59–68)	65 (59–72)	0.301	59 (53–66)	65 (60–73)	0.001
Area under threshold MAP (hours × mm Hg (IQR))									
65 mm Hg	2.0 (0.0–10.2)	0.0 (0.0–3.5)	0.001	1.0 (0.0–6.8)	0.0 (0.0–3.4)	0.159	4.0 (0.0–24.1)	0.0 (0.0–3.5)	0.001
70 mm Hg	10.0 (1.6–29.8)	3.3 (0.0–14.5)	0.001	7.5 (0.8–18.4)	3.3 (0.0–12.4)	0.219	15.5 (4.3–50.8)	3.5 (0.0–15.5)	0.001
75 mm Hg	27.8 (10.9–57.4)	14.4 (0.9–37.0)	0.001	25.5 (5.6–40.0)	15.0 (1.5–33.8)	0.221	39.5 (19.4–78.6)	14.1 (0.8–37.7)	<0.001
Time below threshold MAP (hours (IQR))									
65 mm Hg	0.5 (0.0–3.2)	0.0 (0.0–1.0)	0.002	0.3 (0.0–1.4)	0.0 (0.0–0.9)	0.157	0.8 (0.0–4.0)	0.0 (0.0–1.0)	0.003
70 mm Hg	2.4 (0.8–4.8)	1.0 (0.0–3.0)	0.002	1.8 (0.4–3.3)	1.0 (0.0–2.8)	0.271	2.8 (1.5–5.8)	0.8 (0.0–3.3)	0.001
75 mm Hg	4.6 (2.1–5.7)	2.8 (0.5–5.0)	0.002	3.5 (1.5–5.4)	2.8 (0.5–4.8)	0.263	5.5 (3.4–5.9)	2.8 (0.5–5.2)	0.001

*AKI* acute kidney injury; *IQR* interquartile range, *MAP* mean arterial pressure

**Fig. 2:**
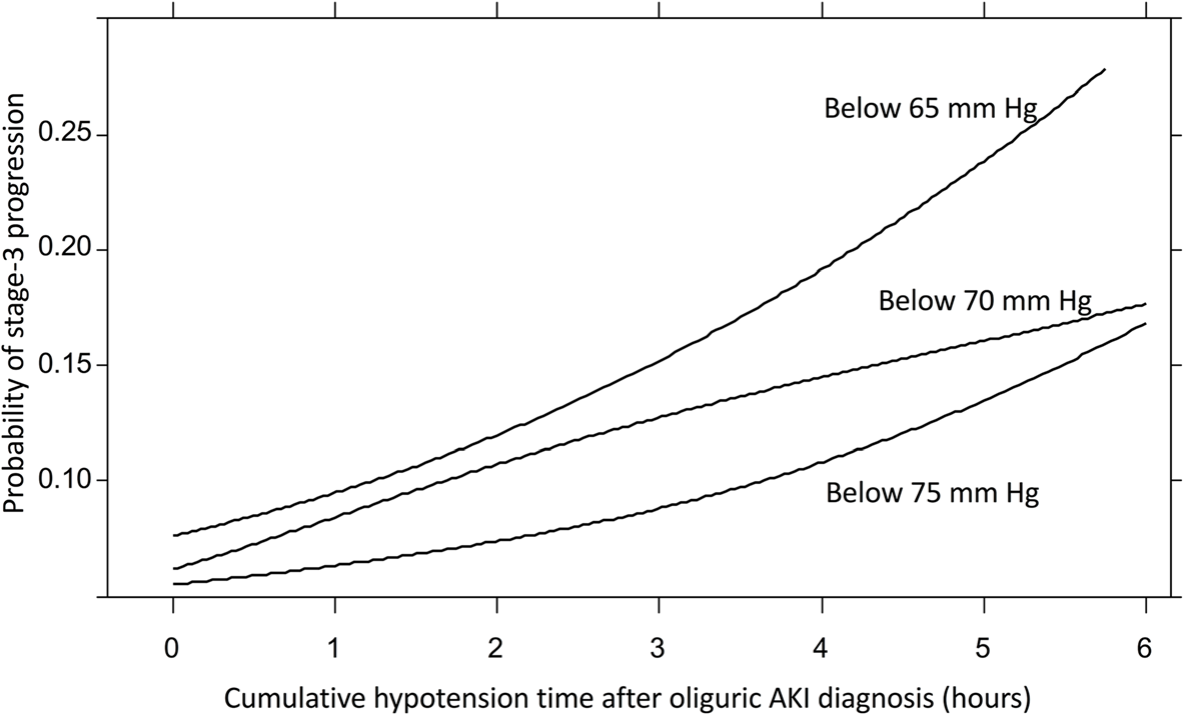
Graphical representation of the relationship between stage-3 progression and cumulative hypotension time below each threshold mean arterial pressure (65, 70, and 75 mm Hg) within 6 h after oliguric acute kidney injury (*AKI*) diagnosis, using restricted cubic splines in univariable logistic regression models

The longer the time below any threshold MAP continued, the more frequently stage-3 progression occurred (Fig. 2 and Table 4). Primary multivariable logistic regression models revealed that a hypotension duration of 3–6 h was significantly associated with stage-3 progression when threshold MAP was 65 mm Hg (adjusted OR 3.73, 95% CI 1.53–9.09, *p* = 0.004); however, such an association was attenuated when the threshold was 70 mm Hg (adjusted OR 2.35, 95% CI 0.96–5.78, *p* = 0.063) and 75 mm Hg (adjusted OR 1.92, 95% CI 0.72–5.15, *p* = 0.200) (Table 4). When the patients were stratified into patients with or without sepsis, the hypotension time of 3–6 h below MAP 65 and 70 mm Hg was significantly associated with stage-3 progression in patients without sepsis (65 mm Hg: adjusted OR 4.53, 95% CI 1.35–15.30, *p* = 0.015; 70 mm Hg: adjusted OR 4.42, 95% CI 1.03–19.00, *p* = 0.046), but was weak and not significant in patients with sepsis (Table 4). The results were similar when we performed sensitivity analyses by focusing on RRT initiation (Table 5).

**Table 4:** Odds ratios of stage-3 progression in time categories below each threshold MAP

	All patients (n = 538)				Sepsis (n = 138)				Non-sepsis (n = 400)			
	Number/total number (%)		Adjusted OR^a^ (95% CI)		Number/total number (%)		Adjusted OR^b^ (95% CI)		Number/total number (%)		Adjusted OR^b^ (95% CI)	
*Stage-3 progression*	54/538	(10.0)	–	–	27/138	(19.6)	–	–	27/400	(6.8)	–	–
Time spent < MAP 65 mm Hg												
None (0 h)	17/266	(6.4)	1.00	Reference	9/65	(13.8)	1.00	Reference	8/201	(4.0)	1.00	Reference
0–3 h	23/216	(10.6)	1.47	(0.71–3.06)	14/60	(23.3)	1.57	(0.60–4.13)	9/156	(5.8)	1.59	(0.52–4.90)
3–6 h	14/56	(25.0)	3.73	(1.53–9.09)	4/13	(30.8)	2.60	(0.61–11.00)	10/43	(23.3)	4.53	(1.35–15.30)
Time spent < MAP 70 mm Hg												
None (0 h)	9/167	(5.4)	1.00	Reference	6/40	(15.0)	1.00	Reference	3/127	(2.4)	1.00	Reference
0–3 h	25/228	(11.0)	1.51	(0.64–3.60)	14/67	(20.9)	1.10	(0.37–3.33)	11/161	(6.8)	3.04	(0.70–13.20)
3–6 h	20/143	(14.0)	2.35	(0.96–5.78)	7/31	(22.6)	1.45	(0.41–5.13)	13/112	(11.6)	4.42	(1.03–19.00)
Time spent < MAP 75 mm Hg												
None (0 h)	6/109	(5.5)	1.00	Reference	3/23	(13.0)	1.00	Reference	3/86	(3.5)	1.00	Reference
0–3 h	14/165	(8.5)	1.53	(0.52–4.48)	10/47	(21.3)	1.86	(0.44–7.99)	4/118	(3.4)	1.13	(0.21–6.12)
3–6 h	34/264	(12.9)	1.92	(0.72–5.15)	14/68	(20.6)	1.42	(0.35–5.88)	20/196	(10.2)	2.78	(0.66–11.60)

^﻿a^Adjusted by Acute Physiology and Chronic Health Evaluation (APACHE) II sores, serum creatinine levels at ICU admission, and prevalence of sepsis. ^b^Adjusted by APACHE II scores, and serum creatinine levels at ICU admission. *AKI* acute kidney injury, *CI* confidence interval, *MAP* mean arterial pressure, *OR* odds ratio, *RRT* renal replacement therapy.

**Table 5:** Odds ratios for RRT initiation in time categories spent below each threshold MAP

	All patients (n = 538)				Sepsis (n = 138)				Non-sepsis (n = 400)			
	Number/total number (%)		Adjusted OR^a^ (95% CI)		Number/total number (%)		Adjusted OR^b^ (95% CI)		Number/total number (%)		Adjusted OR^b^ (95% CI)	
*RRT initiation*	40/538	(7.4)	–	–	24/138	(17.4)	–	–	16/400	(4.0)	–	–
Time spent < MAP 65 mm Hg												
None (0 h)	12/266	(4.5)	1.00	Reference	7/65	(10.8)	1.00	Reference	5/201	(2.5)	1.00	Reference
0–3 h	19/216	(8.8)	1.70	(0.76–3.81)	13/60	(21.7)	1.86	(0.65–5.35)	6/156	(3.8)	1.52	(0.44–5.29)
3–6 h	9/56	(16.1)	3.00	(1.07–8.45)	4/13	(30.8)	3.57	(0.79–16.20)	5/43	(11.6)	2.45	(0.56–10.80)
Time spent < MAP 70 mm Hg												
None (0 h)	7/167	(4.2)	1.00	Reference	4/40	(10.0)	1.00	Reference	3/127	(2.4)	1.00	Reference
0–3 h	19/228	(8.3)	1.42	(0.54–3.69)	13/67	(19.4)	1.55	(0.44–5.50)	6/161	(3.7)	1.28	(0.30–5.47)
3–6 h	14/143	(9.8)	2.05	(0.75–5.64)	7/31	(22.6)	2.34	(0.57–9.53)	7/112	(6.2)	1.73	(0.40–7.41)
Time spent < MAP 75 mm Hg												
None (0 h)	4/109	(3.7)	1.00	Reference	1/23	(4.3)	1.00	Reference	3/86	(3.5)	1.00	Reference
0–3 h	13/165	(7.9)	2.10	(0.62–7.11)	10/47	(21.3)	7.13	(0.78–65.60)	3/118	(2.5)	0.72	(0.14–3.75)
3–6 h	23/264	(8.7)	1.77	(0.55–5.68)	13/68	(19.1)	4.61	(0.52–41.20)	10/196	(5.1)	0.96	(0.24–3.88)

^a^Adjusted by Acute Physiology and Chronic Health Evaluation (APACHE) II sores, serum creatinine levels at ICU admission, and prevalence of sepsis. ^b^Adjusted by APACHE II scores, and serum creatinine levels at ICU admission. *AKI* acute kidney injury, *CI* confidence interval, *MAP* mean arterial pressure, *OR* odds ratio, *RRT* renal replacement therapy.

## Discussion

In this study, we demonstrated that early-phase cumulative hypotension time spent below a particular threshold MAP was associated with progression to stage-3 oliguric AKI (progression to oligoanuria and use of RRT) among critically ill patients with early oliguric AKI, especially in patients without sepsis. This is the first report examining the level and duration of hypotension, and the septic state in patients with early oliguric AKI, and may provide information to guide the management of early-stage AKI in the ICU.

Which blood pressure parameter is the best - time-averaged MAP, the area under threshold MAP, or time below threshold MAP? From the calculated AUROC, the prediction of stage-3 progression was similar. Previous researchers have reported the optimal threshold of blood pressure to prevent AKI by examining time-averaged MAP [4, 5]. However, in our study, although the difference in time-averaged MAP in patients with and without stage-3 progression was statistically significant, we should consider whether the difference was clinically meaningful (71 mm Hg vs. 75 mm Hg) (Table 3). In addition, the method using time-averaged MAP does not consider variation in blood pressure. Is the method appropriate to investigate the optimal threshold of blood pressure? On the other hand, in patients who underwent non-cardiac surgery, the time spent below intraoperative MAP of 55–60 mm Hg has been strongly associated with increased risk of postoperative AKI [8, 9]. The primary outcome in these studies was not progression to severe-stage AKI, and not all patients were admitted to the ICU. Although the outcome and patient profile of these studies are different from those of our study, they indicate the importance of cumulative hypotension time in AKI research. Accordingly, in our study, we examined both area under threshold MAP and time below threshold MAP, considering the importance of hypotension time, and time below threshold MAP seemed comparable with and easier to apply in clinical practice than area under threshold MAP (Table 3).

In this study, hypotension below a particular threshold MAP was associated with stage-3 progression. Surviving Sepsis Campaign Guidelines recommend maintaining MAP ≥65 mm Hg in patients with sepsis [6, 7], while some earlier reports indicate that maintenance of MAP ≥70 mm Hg would prevent AKI and progression to severe-stage AKI. Badin and colleagues reported that time-averaged MAP of 72–82 mm Hg might be necessary for septic shock patients with AKI defined by serum creatinine, to prevent progression of AKI [4]. On the other hand, a recent large randomized controlled trial comparing mortality, AKI incidence, and RRT initiation between the target MAP of 65–70 mm Hg (low-target group) and 80–85 mm Hg (high-target group) in patients with septic shock (the SEPSISPAM study) did not support the maintenance of blood pressure much higher than 65 mm Hg [21]. However, it should be noted that MAP in most patients in the low-target group in this trial was actually maintained at higher than 70 mm Hg [21]. In addition, the target MAP of 80–85 mm Hg in the high-target group might have been much higher than necessary. Therefore, some caution would be needed to interpret the trial results.

Another important result of our study was the association between early-phase cumulative hypotension time and stage-3 progression among oliguric patients with AKI without sepsis, and the association was weak among patients with sepsis in any threshold MAP. In this study, more than 60% of patients without sepsis were postoperative. The mechanism of progression to severe AKI might be different between patients with sepsis and postoperative patients with AKI. Postoperative AKI might be more sensitive to the continuation of hypotension than septic AKI. Factors other than hypotension might affect the stage progression in septic AKI.

Is there a “golden time” to treat early-phase AKI, as in acute myocardial infarction and acute ischemic stroke? We identified an association between cumulative hypotension time and severe oliguric AKI, even in a short time-frame such as 6 h after oliguric AKI diagnosis. A recent study revealed that urine output responsiveness after a furosemide stress test is superior to any recent biomarker in the prediction of severe-stage progression [22]. Another study indicated that oliguric AKI is associated with poor prognosis, even when the serum creatinine level is not increased [23]. These findings, including ours, suggest that urine output might be efficient as a continuous monitor. Early diagnosis of oliguric AKI through continuous urine output monitoring would enable us to initiate earlier treatment of AKI. Effective treatments have still not been established for AKI, but future studies might provide effective procedures including optimal blood pressure levels in patients with early oliguric AKI. If there is a “golden-time” to treat AKI, early diagnosis by urine output and early treatment with blood pressure management would be clinically important.

This study has several limitations. First, the control of confounding factors may be insufficient because of the observational study design. It was difficult to obtain data on diabetes mellitus, chronic hypertension, the presence of hypotension before ICU admission, and the exposure to radiocontrast or nephrotoxic agents. Positive fluid balance has recently been a well-known risk factor for patients’ prognoses [24–26]. In our study, only fluid balance during the first measurable 8-h period after start of oliguria was available. In addition, more than 50% of the included patients were postoperative, but it was difficult to assess retrospectively whether oliguria was due to hypovolemia. Therefore, the results of this study do not directly imply that increasing blood pressure itself has an impact on AKI incidence and progression.

Second, we used only urine output to define AKI and the AKI stages. Therefore, our definition of AKI did not strictly follow the definition in the KDIGO criteria. However, it is well-known that preadmission baseline creatinine data are often unavailable in clinical practice [27]. As shown in our previous paper [19], baseline serum creatinine levels were not known among more than 50% of the patients in the ICU despite an effort to obtain the data. In many AKI studies, the serum creatinine back-estimation method has frequently been used for complementing missing data on baseline serum creatinine [28–30]. However, Bernardi and colleagues have pointed out that this frequently used method, assuming a “true” glomerular filtration rate of 75 mL/min/1.73 m^2^, is not accurate and that it might have caused misclassification [31]. Therefore, although our current study fundamentally targeted only urine output as a continuous monitor, it could be acceptable that we did not use baseline serum creatinine data.

Third, baseline blood pressure measurements could not be obtained in this study. Even in the SEPSISPAM study, which showed no difference in outcomes between the high-target MAP group and the low-target MAP group, the proportion of AKI and RRT among patients with chronic hypertension was lower in the high-target group than in the low-target group [21]. Consequently, baseline blood pressure might be an important co-morbid factor in AKI-related studies.

Last, this study was conducted in a single center, and the number of patients was small. Although sepsis has been reported as the leading cause of AKI in the ICU [1], most patients included in this study were patients without sepsis rather than patients with sepsis, who accounted for only 25.7% of the study sample. Therefore, the generalizability of the study might be limited.

## Conclusions

In conclusion, early-phase cumulative hypotension time below a particular threshold MAP was significantly associated with progression to the severe stage among critically ill patients with early oliguric AKI, especially in patients without sepsis. This association was weak in patients with sepsis. More attention should be paid to the length of time spent in hypotension in ICU care.

## Key messages

- Early-phase cumulative hypotension time below threshold MAP was associated with stage-3 progression of oliguric AKI, especially in patients without sepsis
- The association was weak and was not significant in patients with sepsis
- In future AKI research, it is necessary to further investigate the significance of hypotension duration and the optimal blood pressure threshold

## Abbreviations

AKI: acute kidney injury
APACHE II: Acute Physiology and Chronic Health Evaluation II
AUROC: area under the receiver operating curve
BP: blood pressure
CI: confidence interval
ICU: intensive care unit
KDIGO: Kidney Disease: Improving Global Outcomes
MAP: mean arterial pressure
OR: odds ratio
RRT: renal replacement therapy
